# Assessing the efficacy of male *Wolbachia*-infected mosquito deployments to reduce dengue incidence in Singapore: study protocol for a cluster-randomized controlled trial

**DOI:** 10.1186/s13063-022-06976-5

**Published:** 2022-12-17

**Authors:** Janet Ong, Soon Hoe Ho, Stacy Xin Hui Soh, Yvonne Wong, Youming Ng, Kathryn Vasquez, Yee Ling Lai, Yin Xiang Setoh, Chee-Seng Chong, Vernon Lee, Judith Chui Ching Wong, Cheong Huat Tan, Shuzhen Sim, Lee Ching Ng, Jue Tao Lim

**Affiliations:** 1 Environmental Health Institute, National Environment Agency, Singapore, Singapore; 2 Communicable Diseases Division, Ministry of Health, Singapore, Singapore; 3 Saw Swee Hock School of Public Health, National University of Singapore, Singapore, Singapore; 4 Lee Kong Chian School of Medicine, Nanyang Technological University Novena Campus, Singapore, Singapore

## Abstract

**Background:**

Dengue is a severe environmental public health challenge in tropical and subtropical regions. In Singapore, decreasing seroprevalence and herd immunity due to successful vector control has paradoxically led to increased transmission potential of the dengue virus. We have previously demonstrated that incompatible insect technique coupled with sterile insect technique (IIT-SIT), which involves the release of X-ray-irradiated male *Wolbachia*-infected mosquitoes, reduced the *Aedes aegypti* population by 98% and dengue incidence by 88%. This novel vector control tool is expected to be able to complement current vector control to mitigate the increasing threat of dengue on a larger scale. We propose a multi-site protocol to study the efficacy of IIT-SIT at reducing dengue incidence.

**Methods/design:**

The study is designed as a parallel, two-arm, non-blinded cluster-randomized (CR) controlled trial to be conducted in high-rise public housing estates in Singapore, an equatorial city-state. The aim is to determine whether large-scale deployment of male *Wolbachia*-infected *Ae. aegypti* mosquitoes can significantly reduce dengue incidence in intervention clusters. We will use the CR design, with the study area comprising 15 clusters with a total area of 10.9 km^2^, covering approximately 722,204 residents in 1713 apartment blocks. Eight clusters will be randomly selected to receive the intervention, while the other seven will serve as non-intervention clusters. Intervention efficacy will be estimated through two primary endpoints: (1) odds ratio of *Wolbachia* exposure distribution (i.e., probability of living in an intervention cluster) among laboratory-confirmed reported dengue cases compared to test-negative controls and (2) laboratory-confirmed reported dengue counts normalized by population size in intervention versus non-intervention clusters.

**Discussion:**

This study will provide evidence from a multi-site, randomized controlled trial for the efficacy of IIT-SIT in reducing dengue incidence. The trial will provide valuable information to estimate intervention efficacy for this novel vector control approach and guide plans for integration into national vector control programs in dengue-endemic settings.

**Trial registration:**

ClinicalTrials.gov, identifier: NCT05505682. Registered on 16 August 2022. Retrospectively registered.

**Supplementary Information:**

The online version contains supplementary material available at 10.1186/s13063-022-06976-5.

## Background

In recent years, outbreaks of arboviruses transmitted by *Aedes* mosquitoes, such as dengue, Zika, and yellow fever, have become increasingly common around the world [1]. Key factors for the rise in incidence include increased urbanization, globalization, and long-distance travel, which encourage the spread and endemicity of the viruses and their vectors, particularly the peri-domestic *Aedes aegypti* species [1, 2]. At present, dengue is the most widespread arboviral disease worldwide and is endemic throughout the tropical belt [3, 4]. An estimated 105 million dengue infections occur annually with associated costs of around USD8.9 billion [3, 5]. Other arboviruses that have dominated headlines include Zika and chikungunya: Zika caused a global epidemic in 2016 with more than 1.5 million infections in over 70 countries [6, 7], with transmission still recorded by more than 80 countries in 2019 alone [7]. Meanwhile, chikungunya outbreaks have occurred frequently since the first major outbreak in 2004, which spread from Kenya to Reunion Island, followed by South and Southeast Asia by 2008, and are estimated to have caused an average yearly loss of over 106,000 DALYs globally from 2010 to 2019 [8, 9].

Current treatments for arboviral infections attempt to alleviate the symptoms of infection but do not address the root cause [10]. With the exception of yellow fever and, more recently, dengue, no suitable vaccine is available for arboviruses borne by *Ae. aegypti* [11]. However, vaccination rates for yellow fever are decreasing [12], and the sole licensed dengue vaccine at present, Sanofi-Pasteur’s CYD-TDV, also known as Dengvaxia®, is plagued with safety concerns among immunologically naïve individuals [13]. As such, vector control remains the primary tool for mitigating the spread of arboviruses. Conventional vector control measures include source reduction [14], space spray, and targeted indoor residual spraying with insecticides to kill adult mosquitoes and chemical treatment of containers to kill larvae [15]. These are complemented with enforcement and legislation such as regular home inspections for potential breeding sites, educational campaigns, and community engagement for better environmental management [14, 16]. A major drawback of conventional vector control efforts is the need for thorough and sustained implementation in order to successfully bring down the vector populations [15]. Moreover, vector resistance to insecticides may arise with prolonged use [17]. A recent meta-analysis of conventional vector control measures against dengue vectors showed that their efficacy is limited in some cases [18]. Therefore, there is a pertinent need for novel vector control strategies to mitigate the spread of arboviral diseases.

One novel approach is the incompatible insect technique (IIT), which involves the release of male mosquitoes infected with the maternally inherited intracellular bacterium *Wolbachia* [19–21]. Mating between infected males and uninfected females results in non-viable offspring through cytoplasmic incompatibility (CI), leading to suppression of the mosquito population [20, 21]. *Wolbachia*-mediated IIT as a method of vector control has been tested in China, the USA, Thailand, and Singapore, the country of focus for this study protocol [22–25]. Singapore, among other programs that also attempt to control the *Ae. aegypti* population, has coupled IIT with sterile insect technique (SIT), using irradiation to sterilize residual females (due to imperfect sex sorting) before releases of *Wolbachia-*infected males. IIT-SIT reduces the likelihood of establishment of the released *Wolbachia* strain in field mosquito populations, which would render CI ineffective.

Singapore is an equatorial city-state in Southeast Asia where *Ae. aegypti* is ubiquitous and dengue is hyperendemic [26, 27]. Besides dengue, the country has also reported sporadic chikungunya infections since 2013, following an outbreak in 2008 and 2013 [28, 29] and experienced a Zika outbreak in 2016 [30, 31]. Decades of conventional vector control efforts have contributed to falling dengue seroprevalence rates which has rendered the population vulnerable to dengue outbreaks despite a low *Ae. aegypti* population [32, 33]. The most recent outbreak in 2020 was the largest on record with an all-time high of 1792 weekly reported cases [34], which was partially attributable to non-pharmaceutical interventions motivated by the concurrent SARS-CoV-2 outbreak [35–37].

Singapore has since 2016 been conducting phased field trials to evaluate the use of IIT-SIT to suppress the local *Ae. aegypti* population. IIT-SIT was chosen over an alternative *Wolbachia* dengue control strategy involving introgression of the bacterium into field mosquito populations to reduce their ability to transmit dengue. This latter approach, which requires release of both male and female *Wolbachia*-infected mosquitoes, has shown promise in trials in Australia and Indonesia [38, 39]. However, IIT-SIT harmonizes with Singapore’s decades-long vector control program which focuses on suppressing the local mosquito population. It received greater social acceptance since it does not release biting female mosquitoes and has negligible ecological impact [40]. Additionally, IIT-SIT is not subject to the risk of dengue mutants escaping the inhibitory effect of *Wolbachia* [41]*.* The trials have thus far demonstrated reductions of *Ae. aegypti* populations and dengue incidences by 98% and 88%, respectively [24, 42]. A modeling study has promisingly found that a *Wolbachia*-mediated IIT strategy that was conservatively 40% efficacious would have retrospectively averted an estimated USD330 million in economic costs over the ten-year period between 2010 and 2020 [43].

Building on the success of the phased field studies, the efficacy of IIT-SIT in reducing dengue incidence will next be tested in a national level cluster-randomized (CR) controlled trial by adapting the methods of Anders et al [44]. In this article, we present the protocol of the planned study which, to our knowledge, would be the first CR controlled trial to experimentally measure the efficacy of IIT-SIT in reducing dengue transmission.

## Methods

### Overview

The aim of this study is to determine whether large-scale deployment of *Wolbachia*-infected male *Ae. aegypti* mosquitoes (the intervention, thereafter referred to as male *Wolbachia-Aedes*) can reduce the incidence of dengue in individuals living in intervention (treatment) clusters, compared to individuals living in non-intervention clusters. The two primary endpoints are (1) odds ratio of *Wolbachia* exposure distribution (i.e., probability of living in an intervention cluster) among laboratory-confirmed reported dengue cases compared to test-negative controls, and (2) laboratory-confirmed reported dengue case counts normalized by population size in intervention versus non-intervention clusters. Secondary endpoints include the efficacy of male *Wolbachia-Aedes* deployment in reducing *Ae. aegypti* mosquito populations, the impact of male *Wolbachia-Aedes* deployment on *Ae. albopictus* mosquito populations, and the pre-post trial community attitudes/knowledge and acceptance of male *Wolbachia-Aedes* and other vector control practices. Exploratory endpoints include the impact of male *Wolbachia-Aedes* deployment on the circulation of dengue cases of specific serotypes and on secondary infections.

### Study design

The study is a parallel, two-arm, non-blinded CR controlled trial conducted in Singapore, an equatorial city-state. Fifteen clusters identified as locations at high-risk of dengue transmission were randomly allocated in an 8:7 ratio to receive the intervention or not. These clusters were residential areas with high-rise public housing apartments, with a high risk of dengue transmission, according to a previous study that mapped the spatial risk of dengue transmission in Singapore [45]. The SPIRIT checklist for the study protocol is provided in Additional file 1.

Under the Infectious Diseases Act [46], all laboratory-confirmed cases of dengue are legally mandated for reporting in the national dengue surveillance system. Approval from the Director of Medical Services has been obtained to collect data of dengue-suspected patients, whose blood samples are sent for dengue tests, through a national network of diagnostic laboratories that support private clinics, public polyclinics, or public/private hospitals. Dengue-suspected patients are identified by clinicians through symptoms such as high fever, body aches, and rashes, coupled with absence of respiratory symptoms. Cases and test-negative controls will be retrospectively classified through laboratory test results. In addition, we will also obtain spatially resolved counts of laboratory-confirmed dengue cases reported to the Ministry of Health.

Impact on dengue transmission will be assessed in two primary endpoints. In the first primary endpoint, intervention efficacy will be assessed via a test-negative design comparing the *Wolbachia* exposure distribution among dengue cases to the exposure distribution among test-negative controls. We assume that the relative propensity to seek healthcare for undifferentiated febrile illness at any GP clinic, polyclinic, or public/private hospital in the intervention compared to non-intervention clusters is the same for cases and controls; hence, the distribution of male *Wolbachia-Aedes* exposure in the sampled controls will be equal to the distribution of male *Wolbachia-Aedes* exposure in the underlying source population from which cases arose [44, 47]. The odds of male *Wolbachia-Aedes*-exposure among sampled dengue-positive cases relative to concurrently sampled dengue-negative controls is an estimate of the relative incidence of medically attended dengue in intervention versus non-intervention clusters [44, 47]. Should the relative incidence be 1, there could be said to be null treatment effect in intervention clusters. If male *Wolbachia-Aedes* deployments reduce dengue transmission, the relative incidence of laboratory confirmed dengue cases in intervention versus non-intervention clusters is expected to be less than one.

In the second primary endpoint, intervention efficacy will be assessed by comparing laboratory-confirmed dengue case counts normalized by cluster population size (thereafter also referred to as *incidence rate*) between intervention and non-intervention clusters. Here, we assume a quasi-experimental setting, with parallel trends in the dengue incidence rates for both intervention and non-intervention sites in the pre-intervention period. By further specifying the difference (before/after intervention) in differences (intervention vs non-intervention clusters) in a regression setting, we can estimate a level change in dengue incidence rates in both intervention and non-intervention arms in the post-intervention period, thereby removing other secular factors coincident with *Wolbachia-Aedes* deployments which may have influenced dengue incidence rates independently of *Wolbachia-Aedes* deployments. This strategy provides causal identification of the cluster-level intervention efficacy in reducing dengue incidence through the estimate of the level change.

As described in following sections, intention-to-treat analyses will be performed for both primary endpoints. Where necessary, e.g., in the event of cluster withdrawal, as-treated and/or per-protocol analyses will be performed as secondary analyses.

### Study setting

The study will be conducted in high-rise public housing areas in Singapore. Singapore has an area of 728 km^2^ and a population of approximately 5.7 million as of 2020 [48]. The study sites cover 10.9 km^2^ and have an estimated total population of 722,204, with an average population density of approximately 66,257 persons per km^2^.

Annually, the number of reported dengue cases in Singapore ranged between 51 and 621 cases per 100,000 individuals from 2010 to 2020 [48, 49]. Fifteen clusters were identified as locations at high risk of dengue transmission, each with an average area of 0.79 km^2^ (range 0.53–1.33 km^2^) and 114 high-rise public apartment blocks (range 70–173) [45]. Where possible, manmade or natural borders such as major roads, highways, and water bodies were used to delineate cluster boundaries to limit spillover of male *Wolbachia-Aedes* from intervention to non-intervention clusters, as well as migration of wildtype mosquitoes into clusters. In the absence of such borders, adjacent areas within a 300m radius were designated as buffer release areas, should the cluster be designated for intervention; clusters (inclusive of buffer areas) were kept at least 700 m apart. Conventional vector control activities by public health agencies, such as media engagement, home inspections, breeding site destruction, and space spray in response to dengue cluster, will continue as per routine practice across the study area and duration in both intervention and non-intervention clusters.

### Randomized allocation of the intervention

Randomization was conducted in February 2022. Eight out of 15 clusters were randomly selected to receive male *Wolbachia-Aedes* deployments and the rest designated non-intervention clusters. Due to the small number of clusters available for randomization, selection of clusters relied on a constrained randomization strategy to prevent chance imbalances in baseline characteristics between intervention and non-intervention clusters. The proportion of positive to negative dengue samples was used as the constraining variable. A large number of potential random cluster allocations in 8:7 intervention/non-intervention ratio were generated (*n* = 10,000).

For each allocation, the value of the constraining variable was calculated in each study arm using the aggregate arm-level value. Each potential random allocation was evaluated against the pre-defined balancing criteria (i.e., no statistically significant difference in the proportion of positive to negative dengue samples between the two arms) and removed as a potential random allocation if they were not met. All potential allocations that satisfy the balancing criteria were kept (*n* = 3151, exceeding the threshold of 100–150 allocations recommended in the literature), and a single allocation was randomly selected from within the restricted list of balanced allocations. Finally, a single random draw was used to determine which of the two study arms was to receive male *Wolbachia-Aedes* releases. Randomization will be followed by extensive community engagement to seek the support of local politicians and grassroot leaders of each of the eight intervention clusters.

### *Wolbachia* deployment strategy

Low-dose (~40 Gy or lower) X-ray-irradiated male *w*AlbB-infected *Ae. aegypti* (male *Wolbachia-Aedes*) will be released in designated public locations in high-rise housing estates in the intervention clusters [24]. Releases may commence across all intervention clusters simultaneously or, if operationally more feasible, be staggered between three lots across two months apart. Releases will be conducted twice a week, during periods of highest *Ae. aegypti* activity (weekdays between 0630–1100 h and 1300–1800 h) [24]. To facilitate even distribution of mosquitoes, releases will be conducted in equally spaced release locations per apartment block, on the ground, middle (levels 5–6) and high floors (levels 10–11). Based on previous studies [24], we expect to release 1–6 male mosquitoes per resident, with the release numbers adaptively guided by the Gravitrap index (see below) at each location each release week. Mosquito monitoring, as described in the following section, will continue throughout the study period. Male *Wolbachia-Aedes* will also be released twice a week on the ground floors of high- and low- rise housing estates within the designated buffer areas [50]. Where releases in buffer areas cannot be performed (e.g., schools), releases will be conducted at the boundary between such areas and the intervention sites. This is to counteract immigration of wild-type *Ae. aegypti* females from contiguous non-release areas.

### Entomological monitoring strategy

Gravitraps are simple, hay infusion-filled cylindrical traps with a sticky lining on the inner surface designed to lure and trap gravid female *Aedes* developed by the National Environment Agency, Singapore [51]. Adult *Ae. aegypti* populations in intervention and non-intervention clusters will be monitored weekly using an average of 6 to 9 Gravitraps per high-rise apartment block [24]. Furthermore, screening for the presence of *Wolbachia* in trapped female *Aedes aegypti* may be carried out to monitor for *Wolbachia* establishment*.* While irradiation greatly reduces the risk of establishment, this is a precautionary measure for early detection in the unlikely event that a sufficient number of fertile females are released to result in establishment. If *Wolbachia* becomes established in an intervention cluster, male *Wolbachia-Aedes* irradiated for male sterility at a higher X-ray dose (≥ 40 Gy) or male *Aedes aegypti* infected with another incompatible *Wolbachia* strain may be released to mitigate the establishment, which is expected to hamper mosquito population suppression.

### Data collection, management, and governance

Identifiable individual-level data on laboratory-confirmed dengue cases and dengue test-negative controls (including residential postal codes, basic socio-demographic information, and details about dengue test status) will be obtained from the national surveillance network of diagnostic laboratories which receive samples for dengue testing from private clinics, public polyclinics, and public/private hospitals. Individual-level data will first be cleaned up and checked for duplicates by selected analysts approved to access and handle identifiable data. If test data from more than one episode of febrile illness is reported for a single individual, the data will only be included if the episodes are more than 4 weeks apart. Data will then be de-identified before further analysis. In addition, we will also obtain spatially resolved counts of laboratory-confirmed dengue cases reported to the Ministry of Health. Regular data monitoring will be conducted to identify biases in response and/or missing data.

Access to trial data will require a restricted VPN connection and a data access key only shared with specific individuals authorized by senior management and the trial Principal Investigator. In addition, data can only be accessed by study personnel who have completed all required cyber/data-security training. All patient data, including dengue test status and residential postal code, clinical, and sociodemographic information will be kept strictly confidential. Periodic reviews by data monitors (internal to the National Environment Agency but who are not involved in the trial) will be conducted to ensure adherence to data governance measures.

Trial data and study information will only be released under prior written approval of the Trial Steering Committee. Patient data will not be released without written permission from respective patients, except for monitoring by ethical review board or regulatory agencies in de-identified form. Reporting of study results will not permit identification or the place of residence of individual patients.

### Laboratory investigation

Diagnostic laboratories under the national dengue virus surveillance program use an internally controlled RT-qPCR assay, dengue non-structural protein 1 (NS1) or IgM as diagnostic assays to detect dengue virus in plasma samples from all dengue-suspected patients [44, 52, 53]. We will account for differences in the type of diagnostic tests used during data analysis. On a representative subsample of dengue-suspected patients sent to Environmental Health Institute, IgG in the serum samples will be determined by enzyme-linked immunosorbent assay (ELISA) using the Panbio Dengue IgG Indirect ELISA (Alere Inc., Waltham, Massachusetts). Quality assurance audits are regularly carried out in the laboratories

### Case-control classification

**Fig. 1:**
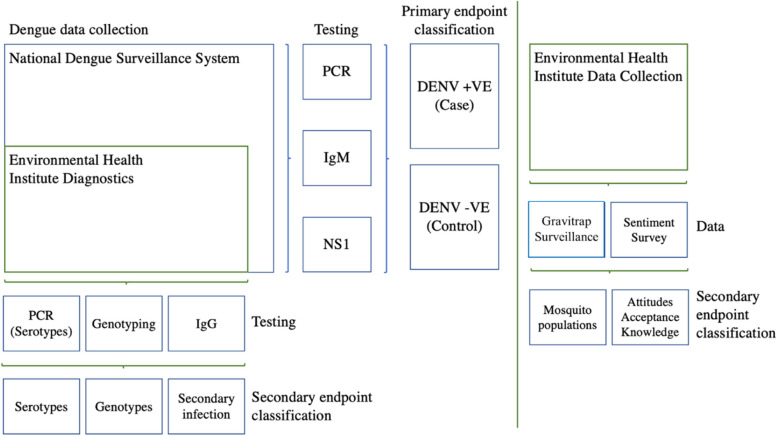
Schematic of case-control classification

Classification of dengue cases and dengue-negative controls is shown in Fig. 1. Dengue cases are patients with virologically confirmed DENV infection through RT-qPCR, testing positive for NS1 antigen or IgM, according to MOH’s criteria. A positive test for any of the three assays would classify the patient as a dengue case. Controls are patients with negative test results for DENV through RT-qPCR, NS1 antigen ELISA, or DENV IgM.

### Expected study duration

Deployment of male *Wolbachia*-*Aedes* and data collection from diagnostic laboratories is expected to commence in June 2022 (see Table 1). Data collection will continue for least 24 months or longer if needed to reach the minimum sample size required for intention-to-treat analysis. To account for year-to-year variability in dengue cases, data collection will continue for at least 24 months even if the estimated minimum sample size is attained sooner.

**Table 1:** Trial timeline

Key activities	Period
Randomization	February 2022
Engage healthcare institutions for data collection [GPs, hospitals, polyclinics]	March 2022–June 2022
Phase 1 sentiment survey	March 2022–June 2022
Community engagement	June 2022 onwards
Start of RCT (releases at 8 intervention sites)	July 2022
Phase 2 sentiment survey	September 2022–December 2022
Phase 3 sentiment survey	December 2022–March 2023
End of RCT	September 2024
Final analysis	September 2024–November 2024
Publication of results	December 2024

### Power calculations—first primary endpoint

It is estimated that 1600 test-negative controls and 400 dengue-positive cases will be needed to detect a 50% reduction in dengue incidence with 80% power. Sample size requirements will be re-estimated using observed data after attaining 50% of the target data collection.

We follow [44, 47, 54] to estimate the sample size for the proposed study design. We used the comparison of exposure odds among test-positive cases vs. test-negative controls for data aggregated across all clusters, with the null hypothesis that the odds of residence in a treatment cluster is the same among test-positive cases as test-negative controls [44, 47, 54]. This provides an unbiased estimate of the relative risk between groups as demonstrated previously [44, 47, 54].

We performed simulations (*n* = 1000) to estimate the power to detect a 50% intervention effect, assuming eight clusters per arm, and using available historical dengue testing data in the 15 study clusters. We randomly allocated eight clusters to receive the intervention; this random allocation was repeated 10,000 times, and allocations were only kept when constrained randomization criterion were met (*n* = 3151 possible balanced allocations). In each iteration of the simulation, we randomly selected one distinct intervention allocation and a 2-year time frame from the historical dengue testing data and simulated a 50% intervention effect in the intervention arm in the latter 12 months. The simulated data was analyzed using logistic regression to assess the significance of intervention effect. These were repeated for various dengue test sample sizes. We computed the proportion of significant results obtained in each scenario. This provided an estimate for the power of our study and the sample size required to obtain the respective power [44].

### Power calculations—second primary endpoint

Intervention efficacy in the second primary endpoint from the difference-in-differences strategy as described below, laboratory-confirmed dengue case counts normalized by cluster population size (incidence rate), which is aggregated at the cluster level and measured across months and across clusters. We followed [55] to estimate the required post-intervention duration required to detect a 50% reduction in dengue incidence rates with 80% power.

The power calculation was conducted by performing simulations (*n* = 1000) to estimate the power to detect a 50% intervention effect, assuming eight and seven clusters in the intervention and non-intervention arm respectively, and using historical dengue incidence rate data (2015–2021) in the 15 study clusters. These clusters were constrained randomized to obtain balanced intervention/non-intervention arms as delineated in the preceding section. In each iteration of the simulation, we randomly selected one distinct intervention allocation and 0.5, 1, 1.5, 2, 2.5, and 3 year time frames post-intervention, with pre-intervention period fixed at 4 years and simulated a 50% intervention effect in the intervention arm in the following intervention. The simulated data was analyzed using the difference-in-differences specification (see below) to assess the significance of intervention effect. We then computed the proportion of significant results obtained in each scenario. This provided an estimate for the power of our study and the measurement duration required to obtain the respective power.

We estimate that at least 31 months would be required post-intervention to detect a 50% reduction in dengue incidence rates at 80% power.

### Pre-post trial attitudes, acceptance, and knowledge survey on *Wolbachia-Aedes* interventions and other vector control practices

To assess efficacy of the various engagement strategies employed, an independent consultancy firm will be engaged to conduct door-to-door household perception surveys. These surveys will be used to measure (1) the effectiveness of different community engagement strategies and (2) each strategy’s efficacy in shaping community attitudes, perception, and knowledge pre- and post-intervention. Surveyors will be trained before data collection to ensure their understanding of the objectives, methodology, expectations, and questionnaires.

The surveys will be conducted in three waves, in households equally selected from the intervention and non-intervention clusters. The first survey will commence three months before and up until the beginning of *Wolbachia-Aedes* deployments. The second and third surveys will begin 3 months and 1 year following the commencement of *Wolbachia-Aedes* deployments respectively. Each survey wave will be split into three arms, (i) non-intervention clusters with no community engagement, (ii) intervention clusters with standard community engagement practices, and (iii) intervention clusters with alternate community engagement strategies. Households will be selected via stratified systematic sampling. Households will be stratified according to ethnic group denomination and minority socio-demographic factors and systemically sampled. Minority groups will be accounted for in higher proportions to reflect adequate representation of these groups. To accurately reflect the general sentiments of the working and non-working populations, surveys will be administered during the evenings of the working days and weekends. The sampled list of households across the different timepoints will be mutually exclusive to prevent contamination of questions across waves. Survey questionnaires used will be tailored to collect specific information across various timepoints and study arms. For each survey session, the surveyor will approach one respondent from each household to interview, after obtaining informed consent to participate. In the event of (i) resident refuses to participate and (ii) resident is not home, the surveyor will note down these households and arrange for a second visit, before approaching the next household on the list. In the event of a language barrier, surveyors will note the language requirements of the household and a second visit with a surveyor equipped with the necessary language skills will return to the household to conduct the survey. A maximum of one revisit to the same household will be attempted to accurately capture ground sentiments, while remaining prudent with resources.

The National Environment Agency’s (NEA) public feedback portal will also be used as an additional data source, to collect residents’ feedback submissions related to *Wolbachia-Aedes* deployment in all clusters. Submissions will be classified as positive or negative and tracked through the duration of the study to supplement understanding of residents’ attitudes, knowledge, and perceptions of the intervention.

### Statistical analysis: primary endpoint—impact of male *Wolbachia-Aedes* deployments on dengue incidence, measured by odds ratio of *Wolbachia* exposure distribution among laboratory-confirmed reported dengue cases compared to test-negative controls

In this analysis, male *Wolbachia-Aedes* exposure will be considered as a binary classification based on whether a patient’s residence is in an intervention cluster or a non-intervention cluster, where residence is defined as the primary place where the patient resided at reporting date. Logistic regression will be used to assess the intervention effect of male *Wolbachia-Aedes* by estimating the aggregate odds ratio, which compares the exposure odds among test-positive cases versus test-negative controls. The null hypothesis is that the odds of residing in the intervention clusters are the same among test-positive cases as test-negative controls.

A secondary cluster-level analysis will be performed using the cluster-level summary measure of the proportion of dengue test-positive individuals among all individuals tested for dengue. The difference in proportions between the intervention/non-intervention clusters will be used to test the null hypothesis of no intervention effect based on the hypothesis tests described in [54]. The average proportions between treatment arms can then be used to infer the relative risk (RR) of dengue in intervention versus non-intervention arms following [44].

### Statistical analysis: primary endpoint—impact of male *Wolbachia-Aedes* deployments on dengue incidence, measured by laboratory-confirmed reported dengue case counts normalized by population size in intervention versus non-intervention clusters

We will monitor trends in reported laboratory-confirmed dengue case counts in intervention and non-intervention clusters, before, during, and after male *Wolbachia-Aedes* deployments take place. We aim to use a quasi-experimental approach to examine the impact of male *Wolbachia-Aedes* deployment on routine dengue case notifications normalized by cluster population size. Namely, we will use regression models to model the monthly, normalized dengue case counts across sites; we will control for population size and seasonal variability through weather station observations and autocorrelation between sites by inclusion of lag terms. To estimate the impact of male *Wolbachia-Aedes* deployments on routine dengue case counts, the regression specification will include a binary group variable to denote whether a cluster is an intervention/non-intervention cluster, and a binary treatment variable denoted 1 will be included to indicate when interventions occur within that cluster [55]. Following similar difference-in-difference strategies [56], the interaction term between the group and treatment variables will then yield a coefficient, which provides an estimate of the intervention effect of male *Wolbachia-Aedes* deployments on monthly reported case counts. This approach ensures that extraneous factors and selection bias, such as level changes in dengue case counts in either intervention/non-intervention arms post-intervention are removed [56]. Should pre-trends assumptions in dengue case counts between intervention/non-intervention clusters be violated under this quasi-experimental approach, we will instead use synthetic control methods which can more appropriately identify the impact of male *Wolbachia-Aedes* deployments on routine dengue case notifications.

In both primary endpoints, intention-to-treat analysis will classify all individuals/clusters respectively allocated to the intervention arm in the randomization stage as such, notwithstanding any non-treatment of the intervention clusters. In per-protocol analysis, any dropouts of the intervention clusters will be removed from the analysis, and only intervention clusters receiving intervention treatment as well as the allocated controls will be analyzed.

### Statistical analysis: secondary endpoint—impact of male *Wolbachia-Aedes* deployments on the prevalence of *Ae. aegypti/Ae. albopictus* mosquitoes

Similarly, we will test whether male *Wolbachia-Aedes* deployments will effectively suppress *Ae. aegypti* mosquito populations as well as whether they have spillover impact on *Ae. albopictus* mosquito populations. Using the nationally representative network of Gravitraps as described above, we will employ a similar quasi-experimental approach to examine the impact of male *Wolbachia-Aedes* deployment on routinely collected *Ae. aegypti/Ae. albopictus* mosquito population data. Namely, we will use regression models with the normal link to model the mean weekly captured *Ae. aegypti/Ae. albopictus* per trap across clusters and control for seasonal variability through weather station observations and autocorrelation between clusters by inclusion of lagged mosquito population terms. To estimate the impact of male *Wolbachia-Aedes* deployments on *Ae. aegypti/Ae. albopictus* mosquito populations, the regression specification will include a binary group treatment variable to denote whether a site is an intervention/non-intervention cluster, and a binary treatment variable denoted 1 will be included to indicate when interventions occur within that cluster [55]. Following other difference-in-difference strategies [56], the intervention term between the group and treatment variables will then yield a coefficient, which provides an estimate of the intervention effect of *Wolbachia-Aedes* interventions on *Ae. aegypti/Ae. albopictus* mosquito populations weekly. This approach ensures that extraneous factors and selection bias, such as level changes in *Ae. aegypti/Ae. albopictus* mosquito populations in either intervention/non-intervention arms post-intervention are removed. Should pre-trends assumptions in *Ae. aegypti/Ae. albopictus* mosquito populations between intervention/non-intervention clusters be violated under this quasi-experimental approach, we will instead use synthetic control methods which can more appropriately identify the impact of male *Wolbachia-Aedes* deployment on the dependent variable of interest.

### Statistical analysis: secondary endpoint—impact of male *Wolbachia-Aedes* deployments on community attitudes/knowledge and acceptance

Using prospectively collected household survey data as described in preceding sections, we will test for differences in engagement and awareness across engagement arms in male *Wolbachia-Aedes* deployment clusters, across 3 sentiment surveys, after which differences and changes in residents’ knowledge attitudes and perceptions on male *Wolbachia-Aedes* deployment and vector control practices will be evaluated across intervention and engagement arms. Responses from all survey waves across intervention/non-intervention sites will first be hypothesis tested for differences in the captured socio-demographic characteristics. If there are between-wave differences in socio-demographic characteristics, propensity score matching will be conducted and/or synthetic weights assigned to each observation to mirror the national level socio-demographic composition. To examine the impact of the trial on community endpoints, logistic/multinomial regressions with site- and trial-specific fixed effects will be used, with socio-demographic controls added. An interaction term between site and trial indicator variables will be added to test overall outcome differences over time between intervention and non-intervention clusters and across engagement arms. Additionally, comparisons across engagement arms will be conducted to determine efficacies of engagement strategies. These analyses will then yield coefficients which allow computation of the odds and marginal odds ratios for each corresponding factor on the community attitude/knowledge/acceptance outcome measure referenced from baseline. Lastly, differences in number of positive and negative feedback submissions through NEA’s public feedback portal will be compared across intervention and non-intervention clusters and across the various engagement arms.

### Statistical analysis: exploratory endpoint—impact of male *Wolbachia-Aedes* deployments on the circulation of dengue serotypes and diagnosed secondary infections

Dengue test samples sent to the Environmental Health Institute will be further sent for serotyping and diagnosis of secondary infections as part of routine dengue surveillance based on the laboratory procedures described in the preceding sections. We will monitor trends in the circulation of specific dengue serotypes and secondary infection cases in intervention and non-intervention clusters, before, during, and after male *Wolbachia-Aedes* deployments have taken place. For assessing the serotype-specific efficacy of *Wolbachia-Aedes* deployments, we will employ a similar logistic regression strategy to estimate the intervention effect of male *Wolbachia-Aedes* by calculating the aggregate odds ratio, which compares the exposure odds among serotype-specific test-positive cases versus test-negative controls (see above).

For assessing the efficacy of *Wolbachia-Aedes* deployments on preventing secondary dengue infection, we will employ the logistic regression to estimate the intervention effect of male *Wolbachia-Aedes* by calculating the aggregate odds ratio, which compares the exposure odds among test-positive cases who are also flagged as secondary infections versus test-negative controls (see above).

### Trial governance and safety

The principal investigator, together with a Trial Steering Committee (TSC) comprising members from NEA, Singapore, and the Ministry of Health, Singapore, will be responsible for ensuring the study is performed in compliance with the approved protocol and the principles of Good Clinical Practice, and will also oversee the coordination of the trial process and data analysis for the trial. The TSC will meet quarterly or as needed throughout the trial. Day-to-day support for the trial, including production, releases, data management, and data analysis, will be overseen by a project team within NEA’s Environmental Health Institute, which meets monthly.

A Review Committee (RC) will be constituted from local and international experts external to the institutions involved in the trial. Its primary role is to ensure the safety and efficacy of the intervention during the trial, as well as overall compliance and conduct of the trial. The RC can provide recommendations to the TSC on continuing/discontinuing the trial and may also make recommendations to the TSC relating to trial procedures, protocol and data management, and quality control as well as analysis. Any proposed major changes to trial protocol will be reviewed by the RC, and approval for a protocol amendment will be sought from the relevant institutional review boards (IRBs) prior to their implementation. Responsibilities and terms of reference will be set out in an RC agreement and agreed to by all RC members prior to study commencement. The RC will meet at study initiation, at 6 months following the commencement of data collection, and at attainment of 50% of the estimated minimum required number of dengue tests (*n* = 1000), as well as any other time at the request of the TSC.

A Data Monitoring Committee (DMC) is not constituted due to the low-risk nature of the intervention. The trial is not considered human biological research, as advised by the Ministry of Health; all laboratory tests will be performed for clinically directed reasons; and data from these tests is routinely collected as part of routine dengue surveillance under the Infectious Diseases Act [46].

### Interim analyses and stopping rules

An interim analysis of the primary endpoints will be conducted when data collection reaches 25%, 50%, and 75% of the estimated minimum required number of dengue tests (*n* = 500, 1000, 1500). Upon interim analysis at the 50% mark, results will be communicated to the RC, and RC may recommend modification/termination of the study if data analysis shows that exposure to male *Wolbachia-Aedes* confers a reduced risk of dengue in the primary endpoints. We follow [44] and will employ the *p* < 0.01 cutoff at interim analysis to be used as guidance for considering early termination. RC may also recommend trial termination if preliminary results at the 50% mark suggest that male *Wolbachia-Aedes* deployments are associated with excess dengue incidence/cases. A less conservative *p* < 0.05 cutoff will be used as guidance for the latter, if results show that the association is in the direction of harm. Termination or modification may also be recommended for any other operational reason (e.g., data collection rates), perceived safety concerns, or external factors. The final decision to terminate or modify the study rests with the TSC.

## Discussion

The *Wolbachia*-based population suppression strategy to reduce *Ae. aegypti* numbers and hence incidence of dengue has been successfully tested in several countries [22, 25]. In Singapore, pilot trials have demonstrated the ability of IIT-SIT to reduce the wildtype *Ae. aegypti* population and dengue incidence in two densely-populated high rise residential estates [24]. In this study protocol, we apply a cluster RCT design comprising both test-negative and reported case count primary endpoints to assess at national level the efficacy of IIT-SIT at reducing dengue incidence. In the context of vector-borne disease control, the cluster RCT and test-negative design have been used in Yogyakarta, Indonesia, to demonstrate the efficacy of *Wolbachia* introgression into field mosquito populations at reducing dengue incidence [39, 44].

Similar to the Yogyakarta study, we apply the CR design which blends elements of the conventional randomized controlled trial (RCT) and the test-negative design (TND) [47, 54]. While a conventional RCT randomizes individuals to intervention or non-intervention groups, a cluster-RCT randomizes clusters of individuals by common identifiers such as spatial location. Meanwhile, the TND is a modification of the case-control design, where cases and controls are recruited from individuals seeking medical treatment because of symptoms consistent with the disease, but not specific to it. Cases and controls are subsequently categorized based on the outcome of diagnostic testing. It was originally meant to address the problem of confounding when cases and controls have different tendencies to seek medical treatment, as both cases and controls would be sampled from the same population that seeks medical care in TND [54].

By blending together the elements of RCT and TND designs, CR-TND is considered a more efficient and cost-effective means of assessing the efficacy of healthcare interventions compared to RCTs and clustered RCTs, provided that several key assumptions are met: (i) the test-negative disease is not associated with the intervention, (ii) the likelihood of seeking treatment is equal between cases and controls in both the intervention and non-intervention clusters, (iii) treatment-seeking behavior is not associated with intervention efficacy, (iv) the diagnostic test used to classify cases and controls is sensitive and specific, (v) controls are sampled from all at-risk individuals in the population without excluding those who have tested positive before, and (vi) data collection on cases and controls occur during the same period when the test-positive disease is present [47].

We also use constrained randomization to allocate clusters to receive intervention or non-intervention. This strategy, which minimizes chance imbalances in baseline characteristics between intervention and non-intervention clusters, is of greater relevance to our study since we have less study sites than the Yogyakarta study, 15 vs. 24 [39, 44]. Singapore is a densely populated city; even though the combined area of our study clusters, 10.9 km^2^, is less than half that of the Yogyakarta study, they contain roughly twice the population and more than 4.5 times the population density. A majority of Singapore’s population reside in high rise public housing apartments similar to those in the study sites. We will deploy male *Wolbachia*-*Aedes* at the ground, middle (levels 5–6), and high floors (levels 10–11) to ensure that the entire block is covered. A previous study has shown that *Ae. aegypti* can be found at all levels of apartment blocks, although lower floors tend to have more mosquitoes than higher floors [57]. We make use of a novel entomological index, the Gravitrap *aegypti* Index (GAI), for entomological surveillance. The GAI normalizes the number of female *Ae. aegypti* caught with the number of Gravitraps in the area and is currently used by the NEA for its vector control operations [57]. Besides epidemiological and entomological endpoints, we will assess the knowledge, attitudes, and acceptance of *Wolbachia*-based vector control practices of the population with two surveys conducted before and after the start of intervention. This is expected to give public health authorities insights into the social acceptability of deploying this strategy at the study sites and help educate the public on the effectiveness of this novel vector control practice.

The effectiveness of the trial will also be assessed via a difference-in-differences identification strategy, using laboratory-confirmed dengue cases normalized by cluster population (incidence rates) as the dependent variable. This quasi-experimental analysis complements the randomized trial design by allowing causal inference of the relationship between *Wolbachia-Aedes* deployment and dengue incidence rates, with the proviso that the pre-intervention dengue incidence trends in both intervention and non-intervention clusters are the same [56]. In view of the limitations in our CR-TND design (elaborated in the next paragraph), such as the relatively small total number of clusters and small area of each cluster, including a quasi-experimental analysis would strengthen our conclusions regarding the causal effectiveness of IIT-SIT at reducing dengue incidence in Singapore.

The protocol has several limitations. Firstly, we cannot guarantee that male *Wolbachia*-*Aedes* will not disperse to non-intervention clusters, especially those released near cluster boundaries. This is particularly relevant given the smaller area of each cluster (range 0.53–1.33 km^2^) compared to the Yogyakarta trial. However, our mark-release-recapture studies found that 90% of male *Wolbachia*-*Aedes* were caught within 40 m of release sites [24], suggesting that the mosquitoes would not disperse too far from their release sites. Secondly, we make the simplifying assumption that all dengue infections occur at the residential addresses of test-positive cases. A related point is that we are unable to prevent changes of addresses by residents from intervention to non-intervention clusters and vice versa during the study, which will affect intervention effect estimates by biasing towards the null. Following [44], we address this issue by powering the study to detect a conservative reduction of dengue incidence of 50%, which accounts for any diminishing of effect sizes due to human movements. The total number of clusters in our trial is relatively small, which is why we adopted the constrained randomization strategy similar to that of [44]. Lastly, we might not be able to obtain data from all diagnostic laboratories in Singapore, which introduces the possibility of confounding if blood samples from dengue-infected cases have different likelihoods of being processed in laboratories not within our network.

The CR-TND design is robust to the patients knowing their exposure status to male *Wolbachia-Aedes*-treatment, as long as this knowledge and any attendant modification of health-seeking behavior apply equally to both cases and controls [44]. Even as the study is carried out, other ongoing strategies to reduce mosquito populations in Singapore will continue as usual, such as inspections to detect hotspots of mosquito breeding and community engagement. We will also monitor for potential field establishment of *Wolbachia*, which would hamper the ability of IIT-SIT to suppress mosquito populations. In the event that establishment is observed, male *Wolbachia-Aedes* irradiated for male sterility or male *Aedes aegypti* infected with another incompatible *Wolbachia* strain will be released to mitigate the establishment.

## Trial status

At the time of submission, the trial has commenced. The current protocol is version 1.0, 1 September 2022.

## Supplementary Information

**Additional file 1.** SPIRIT 2013 Checklist: Recommended items to address in a clinical trial protocol and related documents*.

## Data Availability

Not applicable.
